# Utilization and Experience of an Older Adult Tuition Waiver Program

**DOI:** 10.1093/geroni/igaf122.1271

**Published:** 2025-12-31

**Authors:** Helena Swanson, Andrea June, Erica Dewey, Yein Cho, Cailee Sheehan, Isabella Krumm

**Affiliations:** Central Connecticut State University, New Britain, Connecticut, United States; Central Connecticut State University, New Britain, Connecticut, United States; Central Connecticut State University, New Britain, Connecticut, United States; Central Connecticut State University, New Britain, Connecticut, United States; Central Connecticut State University, New Britain, Connecticut, United States; Central Connecticut State University, New Britain, Connecticut, United States

## Abstract

Outreach and Engagement is a core domain within the Age Inclusivity Domains of Higher Education model. Central Connecticut State University furthers the work of this domain at their Age-Friendly University by advertising and advocating for the use of a tuition waiver available to Connecticut residents aged 62 and older. This waiver allows residents to enroll in courses after minimal fees, assuming open seats in the section and either meeting pre-requisites or obtaining permission from the instructor. While this program has been available for many years, the utilization and experience of those participating has not been explored. Utilization data was obtained with support from the Bursar’s office and the Registrar’s office. Between Spring 2023 and Spring 2025, the program accounted for 1073 credits (61% graduate, 37% undergraduate, and 2% doctoral) across 272 unique individuals. Students were enrolled in courses across the various schools of the campus, though courses in the college of liberal arts and social sciences were more heavily represented. Classes ranged from 1 to 4 credits. More than half of the students (63%) were not pursuing a degree. Only 11 students were enrolled full-time, and all were seeking graduate degrees. Students who have utilized the program were also invited to participate in a semi-structured interview that was recorded and transcribed. Major themes from the interview will be discussed. Taken together, the results of the study will be used to strengthen participation in the waiver program and to provide additional support for the benefits of program to the university.

